# Antibiotic resistance patterns in *Escherichia coli* from gulls in nine European countries

**DOI:** 10.3402/iee.v4.21565

**Published:** 2014-01-10

**Authors:** Johan Stedt, Jonas Bonnedahl, Jorge Hernandez, Barry J. McMahon, Badrul Hasan, Björn Olsen, Mirva Drobni, Jonas Waldenström

**Affiliations:** 1 Centre for Ecology and Evolution in Microbial Model Systems, Linnaeus University, Kalmar, Sweden; 2 Department of Infectious Diseases, Kalmar County Hospital, Kalmar, Sweden; 3 Section of Clinical Microbiology and Infectious Diseases, Department of Medical Sciences, Uppsala University, Uppsala, Sweden; 4 UCD School of Agriculture & Food Science, University College Dublin, Belfield, Dublin, Ireland

**Keywords:** Escherichia Coli, wildlife, birds, gulls, Antibiotic resistance, antibiotics

## Abstract

**Background:**

The prevalence of antibiotic resistant faecal indicator bacteria from humans and food production animals has increased over the last decades. In Europe, resistance levels in *Escherichia coli* from these sources show a south-to-north gradient, with more widespread resistance in the Mediterranean region compared to northern Europe. Recent studies show that resistance levels can be high also in wildlife, but it is unknown to what extent resistance levels in nature conform to the patterns observed in human-associated bacteria.

**Methods:**

To test this, we collected 3,158 faecal samples from breeding gulls (*Larus* sp.) from nine European countries and tested 2,210 randomly isolated *E. coli* for resistance against 10 antibiotics commonly used in human and veterinary medicine.

**Results:**

Overall, 31.5% of the gull *E. coli* isolates were resistant to ≥1 antibiotic, but with considerable variation between countries: highest levels of isolates resistant to ≥1 antibiotic were observed in Spain (61.2%) and lowest levels in Denmark (8.3%). For each tested antibiotic, the Iberian countries were either the countries with the highest levels or in the upper range in between-country comparisons, while northern countries generally had a lower proportion of resistant *E. coli* isolates, thereby resembling the gradient of resistance seen in human and food animal sources.

**Conclusion:**

We propose that gulls may serve as a sentinel of environmental levels of antibiotic resistant *E. coli* to complement studies of human-associated microbiota.

Antibiotic resistant bacteria are a global problem for both human and animal health (1–3). For instance, in Europe, the European Centre for Disease Prevention and Control (ECDC) estimated that the death toll from nosocomial infections with resistant bacteria exceeds 25,000 patients per year (4). The levels of antibiotic resistance have also increased in indicator bacteria from commercially reared swine, poultry and cattle populations in Europe (5), and the resistance levels in indicator bacteria from these food production animals correlate with resistance levels in human samples at the national level, indicating regular transmission of bacteria and resistance genes (6). Furthermore, there are strong indications that the occurrence of resistance in *Escherichia coli* collections from human and veterinary sources correlates with the antibiotic consumption within sampled regions (7, 8). In reaction to this situation, the European Union has evoked measures to reduce unnecessary usage of antibiotics in the member countries (9, 10). For example, since 2006 antibiotics are banned as growth promoters in food production animals ((EC regulation 1831/2003).

The gram-negative bacterium *E. coli* is the most frequently used indicator bacterium for addressing antibiotic resistance dissemination in different environments and host species, as it is a frequent carrier of different antibiotic resistance genes and a prominent constituent of human and most mammal and bird gastrointestinal microbiota (11–13). Based on data from human *E. coli*, a south-to-north gradient in resistance levels has been observed in Europe, with greatest levels of isolates with resistance in the Mediterranean countries and lowest levels in the Nordic countries (14, 15).

Human and food animal bacteria with resistant genotypes are likely frequently disseminated into the environment, via sewage water, for example (16, 17).

In recent years, several reports of antibiotic resistant bacteria from both environmental and wildlife sources have been published (12, 13, 18), and *E. coli* with resistance genes have been isolated from several animal species, including wild boars (19), brown rats (20) and Gilthead sea bream (21). Furthermore, similar *E. coli* sequence types (STs) or clonal groups are often isolated from gulls, humans and domesticated animals (22–25). Generally, in smaller birds, such as passerines, resistant *E. coli* are rare or absent, and studies mainly examining specific resistance markers such as extended-spectrum beta lactamases (ESBL) have been published (26, 27). Larger bird species, especially those that live in close proximity to humans, such as crows, other corvids and gulls seem to be more frequent carriers of *E. coli* resistant to antibiotics common in human and livestock (13). Gulls and corvids utilize food resources made available by humans, and often feed in urban areas, at garbage dumps, at sewage plants or in agricultural areas rich in livestock. As a consequence, resistant *E. coli* can be locally high in some wild bird populations associated with humans (23, 24).

The presence of resistant bacteria in gulls has prompted their use as an indicator of environmental antibiotic resistance occurrence, and there have been several publications presenting data from various gull species in recent years (22–25, 28–33). However, most of these studies have been restricted either in the geographical coverage of the sampling area or in the time span of the investigation, making large-scale inferences and comparisons between studies difficult. In the present study, we sampled gulls in nine different European countries simultaneously during the late part of the breeding season, allowing for comparisons of antibiotic resistance levels in *E. coli* between countries with differing antibiotic resistance levels. As focal species, we used closely related gull species, the Herring gull (*Larus argentatus*) and the Lesser Black-backed gull (*Larus fuscus*) that occur in Northern and Western Europe, and their sister species the Yellow-legged gull (*Larus michahellis*) that occurs in Southern and Southwestern Europe (34). These gulls are widespread, have similar feeding ecology and are common in manmade environments, especially in towns, harbours and areas with trash. Our aim was to investigate the spatial variation in gull *E. coli* resistance levels in a cross-sectional survey across Europe to evaluate the dissemination of resistant *E. coli* in the environment. If the antibiotic resistance levels in gulls are mainly reflecting the situation in the area where they bred, we hypothesize that antibiotic resistance levels in gulls should conform to patterns observed in other sources.

## Material and methods

### Study species, sampling sites and methodology

Sampling was conducted from mid-June to early July 2009, in the late part of the gull-breeding period, where three sampling teams visited eight different European countries, sampling Herring gulls and Lesser Black-backed gulls in northern Europe, and Yellow-legged gulls in southern Europe (Table 1). Additionally, in Ireland one of the authors (BMcM) carried out sampling from June to August. In collaboration with local ornithologists in each country, the field personnel visited localities with large numbers of breeding gulls. From each site, 50–450 samples were collected. Sampling sites were chosen to be as similar as possible between countries, taking human activity and gull feeding habitats into consideration.

**Table 1:** Summary of sampling sites, sampled gull species, number of collected samples, isolated E. coli included in the study and a description of the sites sampled.

Country	Location	Species	Samples *n* (Isolated *E. coli*)	Description
*Denmark*	*Blåvand*	*Larus fuscus (60%)*,	*158 (96)*	*Coastline close to Roskilde*
		*Larus argentatus (40%)*		
*England*	*Bristol*	*Larus argentatus*	*133 (68)*	*Central Bristol*
*Ireland*	*Howth*	*Larus argentatus*	*266 (239)*	*Suburb of Dublin containing fishing harbour*
*Latvia*	*Daugava*	*Larus argentatus*	*323 (224)*	*Close to central Riga*
*Latvia*	*Kaltene*	*Larus argentatus*	*101 (60)*	*Countryside 60 km from Riga*
*Netherlands*	*Borsele*	*Larus argentatus*	*280 (229)*	*Industry harbour*
*Netherlands*	*Rotterdam*	*Larus fuscus (90%)*,	*280 (228)*	*Industry harbour*
		*Larus argentatus (10%)*		
*Poland*	*Wladyslawowo*	*Larus argentatus*	*280 (235)*	*Coastline close to Gdansk*
*Portugal*	*Lagos*	*Larus michaellis*	*314 (176)*	*Fish market in Lagos*
*Portugal*	*Portimao*	*Larus michaellis*	*111 (76)*	*Coastline close to Portimao*
*Spain*	*Emporda*	*Larus michaellis*	*199 (84)*	*City dump outside Emporda*
*Spain*	*Mazarron*	*Larus michaellis*	*321 (200)*	*Breeding colony close to Mazarron*
*Spain*	*Malaga*	*Larus michaellis*	*175 (120)*	*Harbour in Malaga*
*Sweden*	*Hudiksvall*	*Larus argentatus*	*217 (175)*	*Archipelago outside the city of Hudiksvall*

For sampling, a sterile cotton wool swab was swirled in freshly deposited faecal material from the ground where gulls had recently been roosting. To avoid multiple sampling from the same bird, samples were collected from dispersed flocks and always in less numbers than there were individuals present at each site. All swabs were inoculated into tubes containing a bacterial freeze medium (Luria broth; BD, Sparks, USA, phosphate buffered saline containing 0.45% Na-citrate, 0.1% MgSO_4_, 1% (NH_4_)_2_SO_4_, and 4.4% glycerol) and immediately frozen and stored in liquid nitrogen in the field, and later in the laboratory at −80°C. All samples were collected from adult and sub-adult birds in or around breeding colonies.

### Isolation of E. coli and antibiotic resistance testing

In total, 3,158 faecal samples with bacterial growth (101–323 samples/location) were available for analysis (Table 1). Each sample was plated on an Uriselect 4 plate (Bio-Rad Laboratories Ltd, Hemel Hempstead, UK) for isolation of putative *E. coli* and one randomly chosen colony per sample was purified and further isolated. *E. coli* identity was confirmed using conventional biochemical testing (urease-negative, H_2_S-negative, indole-positive and b-galactosidase-positive). After isolation and characterization, 2,210 *E. coli* isolates remained, representing an isolation frequency of 42–90% of the total sample in each country (Table 1).

The susceptibility of each isolate was tested with disc diffusion against a panel of 10 antibiotic agents used in *E. coli* infections in human and veterinary medicine: ampicillin 10 µg/disc, cefadroxil 30 µg/disc, chloramphenicol 30 µg/disc, nalidixic acid 30 µg/disc, nitrofurantoin 100 µg/disc, mecillinam 10 µg/disc, tetracycline 30 µg/disc, tigecycline 15 µg/disc, streptomycin 10 µg/disc and trimethoprim/sulfamethoxazole 25 µg/disc (all antibiotics from Oxoid Ltd, Cambridge, UK). Susceptibility testing was conducted using clinical breakpoints on Mueller-Hinton agar according to EUCAST recommendations, including the *E. coli* ATCC 25922 as a reference strain. Currently, tigecycline and streptomycin lack defined EUCAST breakpoints for susceptibility testing; therefore, breakpoints for these antibiotics were defined according to the NRI method (35).

## Results

### Antibiotic susceptibility of randomly selected E. coli

**Fig. 1:**
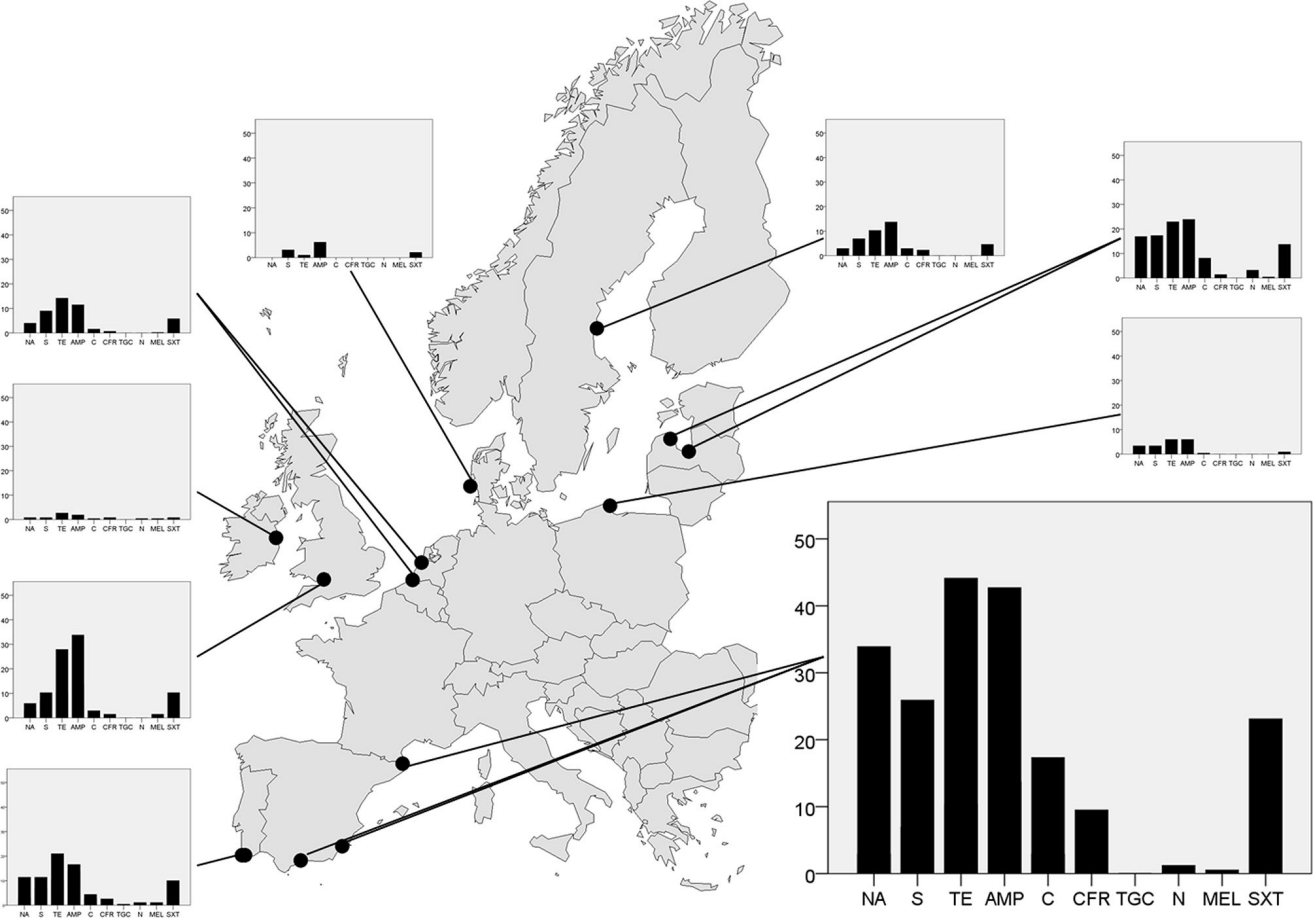
Geographical variation in gull *E. coli* resistance levels in nine different European countries. Dots represent sampling sites in each country, and the bar plots show average resistance levels (%) to each of the analysed antibiotics. The antibiotics, from left to the right: nalidixic acid (NA), streptomycin (S), tetracycline (TE), ampicillin (AMP), chloramphenicol (C), cefadroxil (CFR), tigecycline (TGC), nitrofurantoin (N), mecillinam (MEL) and trimethoprim/sulfamethoxazole (SXT). The bar plot from Spain is enlarged to indicate the scale used.

In total, 31.5% of the 2,210 tested *E. coli* isolates were resistant to ≥1 antibiotic, and 19% of the isolates displayed resistance to ≥2 antibiotics (Table 2). In general, tetracycline resistance was the most common phenotype (19.0%) of *E. coli* isolates, followed by resistance to ampicillin (18.1%), while none of the isolates were resistant to tigecycline, and very few to mecillinam (0.7%) (Fig. 1, Table 3). The level of antibiotic resistance, measured as the number of isolates with resistance to ≥1 antibiotic, varied between countries (*χ* ^2^=626.2 df=8, *p*<0.001), with highest observed levels in Spain (61.2%), England (44.1%) and Latvia (38.7%). Lowest resistance levels were seen in Denmark (8.3%) and Ireland (11.6%) (Table 3).

**Table 2:** The proportion of E. coli isolates with resistance to one or more antibiotics on each sampling site.

	0	≥1	≥2	≥3	≥4	≥5	≥6	≥7	8
Denmark	91.7	8.3	5.2	0.0	0.0	0.0	0.0	0.0	0.0
England	55.9	44.1	25	13.2	7.4	1.5	2.9	1.5	0.0
Ireland	88.4	11.6	1.9	0.4	0.0	0.0	0.0	0.0	0.0
Latvia, Daugava	58.0	42.0	31.7	26.3	18.7	12.5	40.0	0.4	0.0
Latvia, Kaltene	96.7	3.3	0.0	0.0	0.0	0.0	0.0	0.0	0.0
Netherlands, Borsele	75.5	24.5	10.9	7.0	3.5	1.7	0.0	0.0	0.0
Netherlands, Rotterdam	83.3	16.7	9.2	7.4	5.2	2.6	2.2	0.4	0.0
Poland	78.7	21.3	4.7	2.6	1.7	0.9	0.0	0.0	0.0
Portugal, Lagos	76.4	23.6	18.3	11.0	9.4	7.3	2.1	0.0	0.0
Portugal, Portimao	69.1	30.9	24.7	16.0	9.9	3.7	1.2	0.0	0.0
Spain, Emporda	39.3	60.7	40.5	28.6	20.2	10.7	2.4	1.2	0.0
Spain, Mazzaron	32.9	67.1	57.3	45.3	31.5	16.9	5.8	0.9	0.0
Spain, Malaga	50.0	50.0	42.5	35.0	27.5	15.0	7.5	0.8	0.0
Sweden	82.3	17.7	9.7	6.9	4.0	3.4	1.1	0.0	0.0

**Table 3:** Percentage of isolated gull E. coli with resistance to each of the tested antibiotic separated per sampling site.

	Nalidixic acid	Streptomycin	Tetracykline	Ampicillin	Chloramphenicol	Cefadroxil	Tigecycline	Nitrofurantoin	Mecillinam	Trimethoprim/sulfamethoxazole
Denmark	0.0	3.1	1.0	6.2	0.0	0.0	0.0	0.0	0.0	2.1
England	5.9	10.3	27.9	33.8	2.9	1.5	0.0	0.0	1.5	10.3
Ireland	0.8	0.8	2.7	1.9	0.4	0.8	0.0	0.4	0.4	0.8
Latvia, Daugava	21.4	21.4	29.0	29.9	10.3	1.8	0.0	4.0	0.4	17.4
Latvia, Kaltene	0.0	1.7	0.0	1.7	0.0	0.0	0.0	0.0	0.0	0.0
Netherlands, Borsele	2.2	9.6	15.3	13.5	0.4	0.4	0.0	0.0	0.4	5.7
Netherlands, Rotterdam	5.7	7.9	12.3	8.8	2.6	0.9	0.0	0.0	0.0	5.7
Poland	3.4	3.4	6.0	6.0	0.4	0.0	0.0	0.0	0.0	0.9
Portugal, Lagos	12.0	9.4	18.3	15.7	3.7	3.1	0.0	1.0	0.0	8.4
Portugal, Portimao	8.6	14.8	25.9	17.3	4.9	0.0	0.0	0.0	2.5	12.3
Spain, Emporda	32.1	27.4	34.5	39.3	10.7	3.6	0.0	0.0	1.2	15.5
Spain, Mazzaron	36.9	28	52	44.9	20.4	11.1	0	1.3	0.4	29.8
Spain, Malaga	30.8	21.7	37.5	42.5	16.7	10.8	3.3	0.0	0.8	16.6
Sweden	2.9	6.9	10.3	13.7	2.9	2.3	0.0	0.0	0.0	4.6

**Fig. 2:**
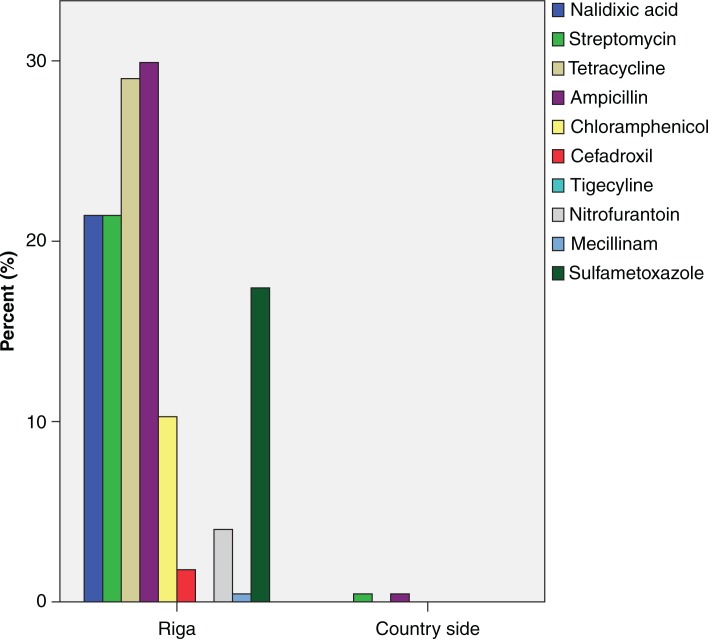
Differences in antibiotic resistance levels between two sampling sites in Latvia. Bars shows the percentage of isolated *E. coli* with resistance to each tested antibiotic.

Similarly, there were also large differences in resistance levels between countries when each antibiotic was analysed separately (Table 3). Greatest resistance levels at the country level were to ampicillin (42.7%) and tetracycline (44.1%) in Spain, but six of the tested antibiotics had resistance levels>15% in at least one country (Table 3). Spain also had the highest proportion (6.5%) of isolates with resistance to ≥6 antibiotics, in contrast to Denmark,Poland and Ireland gull samples that had no isolate with resistance to ≥6 antibiotics (Table 2). In total, 59 different phenotypes with resistance to ≥3 antibiotics were found. At the local scale, the most striking variation in resistance levels was found in Latvia where 224 *E. coli* isolated from a gull-breeding colony situated in the river Daugava, downstream of central Riga, had higher resistance levels than the corresponding 80 *E. coli* isolates from gulls breeding in the countryside 100 km outside Riga (Fig. 2). In other countries with more than one sample site, such as Spain, Portugal and The Netherlands,there were no evident differences in resistance levels depending on sample location.

## Discussion

### Spatial variation in gull E. coli resistance levels across Europe

This study demonstrates that the spread of antibiotic resistant *E. coli* is not confined to clinical and food production environments, as high rates of *E. coli* resistance to ≥1 antibiotic were frequently found in the faecal microbiota of gulls. In six of the countries, >20% of the *E. coli* from the gulls was resistant to ≥1 antibiotic, and in Spain more than half of the isolates had resistant phenotypes. The diversity of multiresistant (≥3 antibiotics) phenotypes within the tested *E. coli* was substantial, with 59 different resistance combinations detected (Table 4). The large diversity in resistance phenotypes both within and between sites indicates that more than one clone per country was involved. High rates of general antibiotic resistance in gull *E. coli* have been observed in previous studies. For example, observed resistance was 47.1% to ≥1 antibiotic in *E. coli* isolated from Yellow-legged gulls in southern France (24). In addition, *E. coli* from Black-headed gulls *Larus ridibundus* sampled in Sweden and the Czech Republic had resistance levels of 13% and 29%, respectively (25, 30). However, our study is the first that investigates the distribution of antibiotic resistance carriage across Europe using a single taxa as a sentinel system.

**Table 4:** All combinations of recognised phenotypes with multiresistance (≥3 antibiotics). Illustrating the diversity of multiresistant phenotypes among the tested E. coli from the nine sampled countries across Europe.

	Poland	Sweden	Latvia	Ireland	England	Netherlands	Spain	Portugal	Denmark	Total
NA-S-TE	0	0	2	0	0	0	2	0	0	4
NA-S-AMP	0	0	3	0	0	0	0	1	0	4
NA-S-C	0	0	0	0	0	0	1	0	0	1
NA-TE-AMP	0	1	5	0	0	3	12	1	0	22
NA-TE-C	0	0	0	0	0	0	0	1	0	1
NA-TE-F	0	0	0	0	0	0	1	0	0	1
NA-TE-SXT	0	0	0	0	0	0	1	0	0	1
NA-AMP-CFR	0	0	0	0	0	0	3	0	0	3
NA-AMP-SXT	0	0	2	0	0	0	1	0	0	3
TE-AMP-C	0	0	1	0	0	0	0	0	0	1
TE-AMP-CFR	0	0	0	0	0	0	3	1	0	4
TE-AMP-MEL	0	0	0	0	1	1	0	1	0	3
TE-AMP-SXT	0	1	0	0	2	1	2	0	0	6
TE-C-SXT	0	1	0	0	0	0	1	3	0	5
S-AMP-SXT	1	0	3	0	1	0	1	0	0	6
S-TE-AMP	1	1	0	0	0	7	8	0	0	17
S-TE-C	0	1	0	0	0	0	0	0	0	1
S-TE-SXT	0	0	1	0	0	1	8	0	0	10
NA-S-TE-AMP	3	0	3	0	0	0	8	1	0	15
NA-S-TE-F	0	0	0	0	0	0	1	0	0	1
NA-S-TE-SXT	0	0	0	0	0	0	2	0	0	2
NA-S-AMP-SXT	0	0	1	0	0	1	4	0	0	6
NA-TE-C-SXT	0	0	1	0	0	0	0	0	0	1
NA-TE-AMP-SXT	0	0	0	0	0	1	7	0	0	8
NA-TE-AMP-C	0	0	0	0	0	0	10	2	0	12
NA-TE-AMP-CFR	0	0	0	0	0	0	2	0	0	2
NA-TE-AMP-MEL	0	0	0	0	0	0	1	0	0	1
NA-AMP-C-CFR	0	0	0	0	0	0	2	0	0	2
NA-AMP-C-F	0	0	1	0	0	0	0	0	0	1
NA-AMP-C-SXT	0	0	0	0	0	0	2	0	0	2
S-TE-AMP-CFR	0	0	1	0	0	0	0	0	0	1
S-TE-AMP-C	0	0	0	0	1	0	0	1	0	2
S-TE-AMP-SXT	0	1	7	0	2	8	12	1	0	31
S-TE-C-SXT	0	0	0	0	0	0	2	2	0	4
S-AMP-MEL-SXT	0	0	0	0	0	0	0	1	0	1
TE-AMP-C-CFR	0	0	0	0	0	0	1	0	0	1
TE-AMP-C-SXT	0	0	0	0	0	0	3	1	0	4
NA-S-TE-AMP-C	0	0	1	0	0	0	4	0	0	5
NA-S-TE-AMP-CFR	0	3	0	0	0	0	1	0	0	4
NA-S-TE-AMP-F	0	0	0	0	0	0	1	0	0	1
NA-S-TE-AMP-SXT	1	1	5	0	0	3	12	10	0	32
NA-S-AMP-F-SXT	0	0	0	0	0	0	1	0	0	1
NA-TE-AMP-CFR-SXT	0	0	1	0	0	0	2	0	0	3
NA-TE-AMP-C-CFR	0	0	0	0	0	0	10	1	0	11
NA-TE-AMP-C-F	0	0	5	0	0	0	0	0	0	5
NA-TE-AMP-C-SXT	0	0	5	0	0	1	1	0	0	7
NA-TE-C-F-SXT	0	0	1	0	0	0	0	0	0	1
S-TE-AMP-C-SXT	0	0	1	0	0	0	6	0	0	7
S-TE-AMP-CFR-SXT	0	0	0	0	0	1	1	0	0	2
S-TE-AMP-MEL-SXT	0	0	0	0	0	0	1	0	0	1
S-TE-AMP-F-SXT	0	0	0	0	0	0	0	1	0	1
TE-AMP-C-CFR-SXT	0	0	0	0	0	0	1	0	0	1
NA-S-TE-AMP-C-SXT	0	2	7	0	1	4	13	3	0	30
NA-S-TE-AMP-F-SXT	0	0	1	0	0	0	0	0	0	1
NA-S-TE-AMP-CFR-SXR	0	0	0	0	1	0	2	2	0	5
NA-S-TE-AMP-C-F-SXT	0	0	1	0	0	0	0	0	0	1
NA-S-TE-AMP-C-CFR	0	0	0	0	0	0	3	0	0	3
NA-S-AMP-C-CFR-SXT	0	0	0	0	0	0	1	0	0	1
NA-S-TE-AMP-C-CFR-SXT	0	0	0	0	0	1	4	0	0	5

NA, nalidixic acid; S, streptomycin; TE, tetracycline; AMP, ampicillin; C, chloramphenicol; CFR, cefadroxil; TGC, tigecycline; N, nitrofurantoin; MEL, mecillinam and SXT, trimethoprim/sulfamethoxazole.

In general, our results conformed to the south-to-north gradient of resistance levels seen in *E. coli* samples from humans and food production animals across Europe (15), but with considerable variation between countries in different regions. For each tested antibiotic, the Iberian countries were in the upper range in levels of *E. coli* with resistance in between-country comparisons, while countries in the northern sampling area (for instance Ireland and Denmark) had a smaller proportion of isolates with resistance. Additionally, antibiotics with overall low resistance levels showed similar geographical patterns. For example, 1.2% of the isolated *E. coli* was resistant to nitrofurantoin in Spain and Portugal, while it was not found at all in Sweden, Denmark, England, Poland and The Netherlands (Table 3). However, the gradient was not absolute, and some countries, such as Sweden and England, had higher resistance levels in the gull samples compared to other countries of the same latitude, suggesting either regional or local variation in dissemination, or sampling effects.

The largest difference among sites within a country was seen in Latvia, where the gull population sampled close to the capital Riga had higher resistance levels than *E. coli* from the population sampled on the countryside (Fig. 2). This suggests that the degree of anthropogenic influence can also vary on a small spatial scale. It also highlights the usefulness of utilizing river catchments and species that use these habitats as sentinels of antibiotic resistance at larger spatial scales. In Spain, Portugal and The Netherlands, where samples were also collected from different locations, we did not detect any significant differences in resistance levels between sampling sites, perhaps due to these sites being more similar in feeding habitats, or human density. We aimed to standardise sampling sites in all countries as far as possible. With the exception of one site in Latvia and, to some extent Sweden, the samples were collected close to larger cities.

Looking at specific resistance profiles, the most frequently recovered phenotypes were resistant to tetracycline or ampicillin. These results are in concordance with other studies on gulls (24, 25, 30) and may reflect the fact that these antibiotics have been commonly used in both veterinary and human medicine for decades. The occurrence of other resistance phenotypes, such as mecillinam and nalidixic acid resistance, was more surprising. Mecillinam resistance is still rare in clinical isolates (36, 37), but was detected in gull *E. coli* in six countries, albeit at low levels. Nalidixic acid resistance has been previously found in *E. coli* from wildlife, for instance, in Yellow-legged gulls (25 and Mallards (31), but the very high rates of resistance to this antibiotic in Spanish gull *E. coli* were remarkable and comparable, or even higher, than the levels seen in human clinical isolates in Spain (36, 38).

Tigecycline was the only tested antibiotic that we did not find any resistance to. It is a relatively new antibiotic, used for skin-structure infections and complicated intra-abdominal infections, and has a broad spectrum activity against multidrug-resistant Gram-positive and Gram-negative pathogens; consequently, the resistance levels to tigecycline are still low in human populations (39).

### Origin of resistance?

There are comparatively few studies that present data on general resistance levels from *E. coli* isolates at larger spatial scales in Europe. EFSA publishes a yearly report on resistance data for bacteria from food production animals that covers large parts of Europe. A recent study compared resistance in human *E. coli* from the EARSS scheme with EFSA *E. coli* resistance data from food production animals, which showed that resistance levels in food production animals (especially poultry and swine) *E. coli* and human *E. coli* correlated at the country level (6). A key issue for the future is to increase monitoring schemes with standardised methodologies for measuring and interpreting antibiotic resistance in bacteria from humans, food production animals and the environment. In this regard, EFSA already has an ongoing harmonised monitoring system (40), while suitable reference material of single antibiotics from human indicator bacteria is lacking. Without such baseline data, our understanding of antibiotic resistance dissemination could be obscured.

From available data, it has been shown that Sweden and The Netherlands have similar low antibiotic usage in the human health care sector (14), and also a comparable low resistance level among *E. coli* from humans (15). However, antibiotic usage in food production is much higher in The Netherlands, and concomitant high resistance levels are seen in *E. coli* from Dutch food animal sources (5, 41). It is interesting to note that this pattern was also seen in the gull samples, where resistance levels to single antibiotics were higher in The Netherlands than in Sweden for five antibiotics, including tetracycline and streptomycin, while Sweden only had higher levels to cefadroxil and chloramphenicol. These differences indicate that food production could be important for the dissemination of resistant bacteria to the environment. However, at the same time, The Netherlands has a far greater population density than Sweden, thereby potentially increasing the likelihood of transmission from human or food-producing animals to wildlife, which could further disseminate resistance. The mechanisms of how gull behaviours and diets vary at the local scale and the links this may have for exposure to resistant bacterial isolates from either human or food animal origins have yet to be demonstrated.

Based on the findings of this and previous studies, we propose that gulls could be used as a sentinel of environmental levels of antibiotic resistance. Gulls are distributed in most parts of the world, enabling comparisons on a local scale, as well as between countries. Several studies have shown that gulls frequently carry resistant *E. coli* (24, 30), often with human-associated genotypes (22, 24). Importantly, the *E. coli* resistance patterns observed in the breeding gulls in this study correlated with data from humans and food production animals sampled in the same regions. Given the between-site variation, comparisons should preferably be performed between areas that have similar human density and feeding habitats for gulls. Through their feeding ecology, gulls are prone to interact with waste products from humans and food animal origin. Gulls often congregate in flocks for foraging, in fields, dumps and harbours where it is relatively easy to collect faecal samples. However, some caution is advisable: many gull species are migratory and birds within a flock may therefore have originated from different locations, obscuring the link between gulls carrying antibiotic resistant bacteria and a particular region, and sampling should therefore be performed during breeding times prior to migration. An important question to ask is for how long do gulls shed resistant bacteria, as this figure would influence the potential of birds introducing resistant bacteria to new areas far from the origin. For *Salmonella*,the shedding time in Herring gulls is short, only a few days (42), while some empirical data suggest that *Salmonella* can survive in the soil of gull nesting colonies between breeding seasons (43). Furthermore, chickens can carry resistant *E. coli* for several weeks (44), and it has been suggested that gulls and other birds bring antibiotic resistant bacteria to very remote areas in the Arctic, indicating longer shedding time (22, 45).
